# Perinatal Maternal Mental Health, Fetal Programming and Child Development

**DOI:** 10.3390/healthcare3041212

**Published:** 2015-11-26

**Authors:** Andrew J. Lewis, Emma Austin, Rebecca Knapp, Tina Vaiano, Megan Galbally

**Affiliations:** 1 School of Psychology, Faculty of Health, Deakin University, Melbourne 3102, Australia; E-Mails: eeaust002@myacu.edu.au (E.A.); rknap@deakin.edu.au (R.K.); TVaiano@mercy.com.au (T.V.); 2 Centre for Social and Early Emotional Development, Deakin University, Melbourne 3102, Australia; 3 Perinatal Mental Health Unit, Mercy Hospital for Women, Heidelberg 3084, Australia; E-Mail: MGalbally@mercy.com.au; 4 Department of Obstetrics and Gynecology, Faculty of Medicine, University of Melbourne, Melbourn 3052, Australia

**Keywords:** fetal programming, perinatal depression and anxiety, mental health in pregnancy, child temperament, growth, cortisol, placenta, epigenetics

## Abstract

Maternal mental disorders over pregnancy show a clear influence on child development. This review is focused on the possible mechanisms by which maternal mental disorders influence fetal development via programming effects. This field is complex since mental health symptoms during pregnancy vary in type, timing and severity and maternal psychological distress is often accompanied by higher rates of smoking, alcohol use, poor diet and lifestyle. Studies are now beginning to examine fetal programming mechanisms, originally identified within the DOHaD framework, to examine how maternal mental disorders impact fetal development. Such mechanisms include hormonal priming effects such as elevated maternal glucocorticoids, alteration of placental function and perfusion, and epigenetic mechanisms. To date, mostly high prevalence mental disorders such as depression and anxiety have been investigated, but few studies employ diagnostic measures, and there is very little research examining the impact of maternal mental disorders such as schizophrenia, bipolar disorder, eating disorders and personality disorders on fetal development. The next wave of longitudinal studies need to focus on specific hypotheses driven by plausible biological mechanisms for fetal programming and follow children for a sufficient period in order to examine the early manifestations of developmental vulnerability. Intervention studies can then be targeted to altering these mechanisms of intergenerational transmission once identified.

## 1. Introduction

Maternal mental disorders are known to be a major complication of pregnancy [1] and there is already substantial evidence from cohort studies that there are important implictions for child development [2,3]. However, the specific mechanisms driving poor child outcomes are not well understood. Since such child outcomes are in no way inevitable, it is also important to understand the specific circumstances where risk is exacerbated or attenuated [2]. Researchers in mental health sciences are increasingly making use of the Developmental Origins of Health and Disease (DOHaD) model to understand how maternal mental disorders in pregnancy impact on the intrauterine environment and influence fetal development. Identifying the specific features of fetal development which predict child and adolescent mental health outcomes will inform perinatal treatment options and very possibly major prevention opportunities that can be delivered in pregnancy [4,5].

In this paper we focus on several biological processes—hormonal and epigenetic programming, and placental functioning—and present evidence from studies indicating their role in the transmission of maternal mental health. Here we limit our focus to a brief and selective review of three developmental outcomes: fetal growth, child temperament, and child and adolescent mental health.

## 2. The Prevalence of Mental Disorders and the Complexity of Exposures in Pregnancy

Psychiatric symptoms in the perinatal period are diverse, and vary from the mild and transitory symptoms of so-called “baby blues” through to postpartum psychotic conditions which are rare and affect less than 1% of the population. Of course, many women also bring into pregnancy pre-existing psychiatric disorders. Perinatal depressive and anxiety disorders are the most commonly researched mental disorders across pregnancy [6,7]. In Australia approximately 16% of women experience depression in the postnatal period and 9% in the antenatal period [8] however, other studies suggest that rates of depression are higher in pregnancy than in the postpartum period [9]. There is considerable continuity of depressive symptoms from pregnancy into the postpartum period, with around 50% of women with postnatal depressive symptoms having also experienced depression during their pregnancy [10]. The rate of maternal anxiety in the antenatal period is less well researched, with some studies reporting prevalence to be approximately 7% [11]. There is a high rate of comorbidity between different antenatal mental disorders [12] potentially confounding investigations into the effect of any single mental disorder on fetal development.

There is considerable complexity arising from high co-morbidity across mental disorders in pregnancy. There is also a wide range of pregnancy exposures that often co-occur in mothers with mental disorders, complicating investigations in fetal development. Women depressed in pregnancy have been found to be more likely to smoke cigarettes [13], a factor shown to be associated with reduced birth weight and with consistent epigenetic changes in offspring tissue [14,15]. A higher proportion of depressed women also use alcohol and illicit substances throughout pregnancy and this is associated with a range of adverse outcomes for the fetus [16]. Maternal alcohol use during pregnancy increases the risk of pre-term birth [17], alters the pattern of fetal and childhood growth [18] and in extreme cases causes congenital abnormalities [19]. Antenatal maternal cannabis use has also been found to impact on aspects of fetal development such as growth restriction and, in high levels of exposure, produces features similar to fetal alcohol syndrome [20].

Women who are depressed in pregnancy are more likely to use psychotropic medications [13] and this adds major complication to research into fetal exposures [21,22,23]. To date the most comprehensive investigations have been undertaken examining antidepressant use in pregnancy however, it is unclear whether this is associated with effects on fetal development either in terms of reducing depressive symptoms and thereby reducing any negative impact of maternal depression on the fetus, or conversely, showing subtle adverse effects on fetal development [24,25,26]. Given the increasing use of antidepressants during pregnancy to treat maternal depression and anxiety [27], the influence of antidepressants on fetal development and child outcomes is of particular clinical interest [13,26].

Poor maternal nutrition has also been associated with abnormal fetal development. This includes effects of iron deficiency, low vitamin D, as well as oxidative stress and chronic inflammation associated with poor diet [28,29]. Poor maternal nutrition can result in abnormal growth and development of the placenta and impaired transference of nutrients from the maternal placenta to the fetus [30]. Maternal body mass index is also a likely influence on fetal development [31], however, to date only four studies have examined the interaction between maternal mental health, fetal development and maternal BMI [32]. In this context it is surprising that so few studies have examined the likely interactions between psychological distress and maternal nutrition or lifestyle factors during pregnancy. These are particularly pertinent when considering that dietary and lifestyle enhancement are highly feasible and promising interventions which may even preserve optimal fetal development in the context of maternal psychological distress, although such approaches require further development and evaluation [33].

## 3. DOHaD and Fetal Adaptation

From a child development perspective, the DOHaD model challenges and extends the traditional psychological framework which usually conceptualises child development as consisting of interactions between so-called innate factors and the influence of postnatal “environmental” factors [34]. Postnatal factors are often considered to be the child’s social, family, peer or school environment. Instead, the DOHaD model proposes that *in utero* development and possibly even factors prior to conception such as the mother’s pre-pregnancy weight, nutritional status or her mental health history can influence maternal functioning over pregnancy and in this way can be transmitted to the child during fetal development. Although it has its origins in epidemiological studies, one of the hallmarks of DOHaD is the strong focus on the mechanisms driving fetal and early postnatal development. The framework integrates evolutionary theory, molecular sciences and draws on experimental animal models to test causal pathways [5]. The DOHaD framework is therefore an important supplement to existing concepts used in developmental psychopathology such as developmental lines of continuity and discontinuity, resilience and vulnerability to adverse exposures, moderation of outcomes via genetic factors, cumulative and interactive combinations of risk factors and kindling effects across development [33,35]. Although beyond the scope of the current paper, a comprehensive theoretical integration of DOHaD concepts into the wider mental health sciences would greatly advance empirical work seeking to understand the earliest vulnerabilities for future mental disorders.

There is a long tradition of speculation that perinatal events could influence an individual’s health throughout their life. David Barker’s epidemiological studies in the 1980s began with the discovery of geographic associations between infant mortality rates and later mortality due to ischemic heart disease in areas of England and Wales [36]. This was followed by the examination of birth weight recorded in Hertfordshire in the early 20th century linked to records of mortality rates from ischemic heart disease in adulthood. Further studies showed similar associations between birth weight and impaired glucose tolerance, type II diabetes and metabolic syndrome. The compelling weight of evidence, replicated across numerous cohort studies and data linkage studies, suggested an alternative to genetically or environmentally determinist models, and instead that a developmental-evolutionary model of disease was needed. The DOHaD model was initially focused on low birth weight which was considered to be a robust marker for the health of the intrauterine environment [36] and the so-called Barker hypothesis proposed that the quality of the intrauterine environment was a major factor in future health and disease arising from the programming effects of maternal pregnancy nutrition [5].

The conceptual basis of the DOHaD model draws heavily on a synthesis of both developmental and evolutionary concepts [37]. Firstly, the fetus is considered to be developmentally plastic and be capable of using a variety of mechanisms to adapt to its intrauterine environment [5]. Developmental plasticity is an evolved trait, but developmentally, it is likely the degree of plasticity will vary across the life course. In species like humans, with long and complex life histories, early fetal and neonatal development is likely to be a period where the developing organism is open to environmental information while later childhood may be a period of being less open to environmental information [38]. Further, developmental plasticity is expected to vary according to the nature of the environmental input. Namely, the capacity of the organism to alter its developmental course is most likely to be highest for inputs which are evolutionarily predictable. So it follows that developmental plasticity should be high to exposures such as stress, variation in nutrition and effects of parity. However, novel exposures (from an evolutionary perspective) such as neurotoxic exposures to smoking or even early weaning would be predicted to induce greater physiological effect and alter the developmental pathway to a greater degree since fetal response is likely to be less adaptive. For example, in a nutritionally sparse environment, the fetus shows remarkable developmental plasticity in being able to preserve brain and heart vasculature at the expense of other organ growth. However, in response to exposure to maternal smoking and even second hand smoke from paternal smoking, fetal growth is significantly compromised and there are long lasting developmental impacts [39].

Another feature of the DOHaD model of development is the idea imported from Life History Theory that there are two forms of adaptation: immediate adaption to environmental demands and a mode of predictive adaptation to expected future environments. Development has a forecasting aspect whereby an organism is seeking to predict the kind of environment it is likely to encounter in its future. Fetal development is seeking to optimize not merely survival but also the more general goal of reproductive fitness and comes to the fore particularly during the pubertal transition. However, once in train, the developmental life course cannot easily be reversed. What may be adaptive changes of the fetal endocrine-metabolic systems *in utero*, may predispose the adolescent or adult to vulnerability to biological or psychosocial challenges. The onset of Common Mental Disorders such as depression and anxiety provide some of the most compelling examples of this process as we outline below.

## 4. Hormonal Programming

Programming refers to the influence of specific exposures that trigger biological processes operating during critical periods in early life, which in turn produce stable and long term alterations in the organism’s developmental pathway [38]. Within a health and medical context, the concept of programming has been proposed to provide an explanatory framework for understanding observed associations between environmental exposures in early development and later disease, or psychiatric disorders in the mature organism. Thus, programming effects refer to the specific influence of a specific factor during a well-defined developmental period as it influences the organization of target tissues or gene-expression patterns that, in turn, influence the course of development and functioning across the life course.

Cortisol provides a good example of hormonal imprinting where exposure of the fetus to maternal cortisol during fetal development may impact on the responsivity of neuroendocrine systems related to stress and psychopathology in later development. Specifically, HPA-axis reactivity can be altered over the long term either in terms of baseline levels, diurnal patterns or degree of response to acute stress. The exact mechanisms involved are complex but the effect might occur in relation to establishing the density of hormone receptor sites such as the glucorticoid receptor in target brain regions such as the hypothalamus or hippocampus. Variation in HPA activity may be due to changes in the activity or concentration of glucocorticoids or it may be due to alteration in glucocorticoid receptor density in target tissues or even to alteration in cortisol metabolism [40]. Variation in the density of receptor sites is likely to have long-term functional implications. An example here is work done largely in rodent models showing that stressful early life exposures induces differences in the degree of methylation in glucocorticoid receptors in the hypothalamus with functional impacts on later stress related behaviour [41].

Also in animal studies, the phenomenon of maternal prenatal priming of postnatal maternal behaviour offers a further example of hormonal programming. In normal development, increases in oestrogen over pregnancy prime oxytocin receptors in the limbic system, reducing maternal fear of novel stimuli and increasing the mother’s receptivity to the otherwise novel social signals coming from the infant. In turn, post-natally these infant signals and behaviour such as nursing trigger additional maternal affective and motivational systems, underpinned by endocrine changes, resulting in sustained parental investment. This particular model has yet to be fully tested in humans although there are a number of promising studies (See review by Galbally *et al*. [42]).

## 5. Epigenetic Programming

Epigenetic factors are non-genetic factors which regulate genomic activity across development and are also known to operate across generations as a non-genomic mode of inheritence. Epigenetic processes do not change the DNA nucleotide sequence but can modify the pattern of transcription of genes in a tissue or cell specific manner [43]. This includes DNA methylation and the acetylation and methylation of histones that influence chromatin structures [44]. Changes in these epigenetic markers have been shown to be associated with exposures such as stress, depression, diet, obesity, smoking and postnatal caregiving although in many cases the direction of causality has yet to be established. Both DNA methylation and histone modification play a fundamental role in differentiation of cell structure and function during embryogenesis and fetal development. As such, epigenetic studies of early development have become a cornerstone of DOHaD research and offer great promise of pointing towards key biological mechanisms following deleterious exposures and driving poor child health and mental health outcomes.

Emerging evidence suggests that epigenetic programming continues with significant dynamism across early postnatal life but it is generally accepted that the methylation status of many genes is established in very early development. Altered epigenetic profiles may mediate links between specific intrauterine and early postnatal exposures and future mental health outcomes [38]. As noted by Meaney the recent discovery of the extent to which environmental signals can remodel epigenetic markers represents a major conceptual shift in the understanding of environmental influence on gene expression and function [41].

However, this science is in its early days and there remain major questions in the epigenetic field. These include the developmental periods of greatest susceptibility to modification, how genetic variation may affect epigenetic modifications, the relative stability of methylation in functional sites and which cell types are most closely related in their epigenetic profiles. Above all, clinicians are interested in whether environmentally induced epigenetic changes can be reversed with early interventions.

A good example of epigenetic influences on fetal development is the case of maternal smoking during pregnancy. While there is plenty of evidence that maternal smoking and even second hand smoke exposure in pregnancy has a range of negative effects on fetal and later child development, epigenetic studies have pointed to a very specific marker of such exposure in the child’s epigenome. There is now consistent evidence of epigenetic modifications consistently associated with the offspring of maternal smokers with several studies now reporting hypomethylation of the *AHRR* gene in neonatal blood [15]. There is also preliminary evidence that these epigenetic changes due to fetal exposure to maternal smoking may even transmit to the grandchild generation.

There are a wide range of pregnancy exposures that often co-occur and complicate investigations of any single exposure on fetal and then child development. As mentioned above, smoking in pregnancy is linked to depressive symptoms, more frequent use of psychotropic medications and other prescribed medications, [13] poor maternal nutrition, iron deficiency and low vitamin D [29]. Carefully designed epigenetic studies may well reveal that each of these exposures is associated with a specific epigenetic biomarker during fetal development and, in turn, these markers may predict future disease and disorder.

## 6. Placental Programming

The placenta serves as an interface between maternal and fetal physiology and functions as a temporary endocrine structure, which regulates the transfer of nutrients to the fetus and protects it from the growth-inhibiting effects of maternal glucocorticoids. There is increasing interest in the role placental biology might play in the interface between maternal antenatal distress and fetal development.

One area of focus has been fetal exposure to glucocorticoids, be they of maternal, placental or fetal adrenal origin. The availability of cortisol to the fetus is regulated by the *11β-HSD* isozymes. This enzyme is highly expressed in the placenta and the *11β-HSD2* form inactivates maternal cortisol during its passage into the fetal blood stream. Decreases in placental *11β-HSD2* would be expected to increase the fetal circulation of glucocorticoid with a range of impacts on fetal development including restriction of fetal growth and particularly head growth, fetal HPA axis development and the hormonal programming of neuroendocrine aspects of the stress response. These aspects of fetal development may well have long-term implications for vulnerability to a range of cognitive and emotional disorders, particularly depressive, anxiety and attention related mental health conditions. In this way the expression level of *11β-HSD2* in the placenta directly influences the exposure of the fetus to circulating maternal stress hormones. Evidence that maternal depression or high stress during the antenatal period can lead to a down-regulation of *11β-HSD2* suggests that it may be a key regulator of infant growth, development and stress response playing a potentially critical role in the formative stages of the fetal HPA-axis. Animal studies have shown a link between maternal stress and placenta 11β-HSD2 activity and a small number of recent findings indicate that there may be a similar pattern evident in humans [45] although further work is required.

## 7. Maternal Mental Health and Fetal Programming

Maternal psychological distress is accompanied by metabolic and functional changes that may also influence fetal development. These include autonomic changes, disturbance of maternal circadian rhythms, and behavioral changes that may influence maternal diet and lifestyle. Such physiological changes may influence the availability of oxygen and glucose to the fetus, disrupt maternal and placental endocrine functions, induce fetal oxidative stress, or reduce fetal circulation of insulin like growth factors (IGF I and IGF II) which directly regulate fetal development and growth [46]. As Barker (1997) [47] has argued, fetal adversity, even for a short period can slow and alter the patterns of rapid cell division in fetal development, either as a direct effect of under-nutrition or via changes in the function of growth factors or hormones. Neural development can similarly be impacted, affecting not only cell density and function but the developing connectivity across neural regions [48]. It is possible that these processes result in not only intrauterine growth restriction or preterm birth, but in long lasting effects on mental health via fetal HPA axis programming [30]. There are a variety of mechanisms by which maternal mental disorders may impact on fetal development. In most, but not all studies, maternal psychological distress is associated with elevated maternal stress hormones such as cortisol, adrenocorticotropic hormone (ACTH) and adrenaline [49].

Alternations in maternal levels of these hormones may be transferred to the fetus via the placenta [50]. However, it is known that maternal circulation of glucocorticoids naturally increases across pregnancy [51] and the placenta may have adaptive mechanisms designed to protect the fetus from these maternal changes (as mentioned in the previous section). One study has found that corticotropin-releasing hormone levels (CRH) in mid-pregnancy are associated with prenatal, but not postnatal depression but overall the literature on maternal mental health and CRH in pregnancy shows mixed findings. In short, any transmission of maternal mental state during pregnancy must be traced to alterations in maternal physiology, and via the placenta which may moderate, adapt to, or exacerbate maternal conditions, onto the fetus [52]. It is also likely that the developing fetus exerts an influence on maternal mental state, equally via placental passage.

## 8. Child Outcomes following Maternal Mental Disorder in Pregnancy

Traditionally one of the key motivations in perinatal mental health for addressing maternal depression and anxiety was not only to address the mother’s psychiatric disorder but to also improve the quality of the parent-child relationship. However, The DOHaD model draws attention to the role of antenatal mental health and the findings emerging from fetal programming research that suggest that the basic platform for the child’s future stress biology is probably being established during the intrauterine period. The DOHaD model implies that prevention interventions ought to be focused on preconception and pregnancy mental health and exposure of mothers to acute or chronic stressors over pregnancy. Early exposure of offspring to maternal anxiety and depression has been associated with vulnerability to behavioural and emotional problems during childhood and adolescence [33]. Maternal stress experienced during pregnancy is associated with pre-term delivery [53], later symptoms of ADHD and delayed language development during childhood in several studies. Maternal prenatal cortisol concentrations have been examined extensively as a predictor of child developmental outcomes and these associations have been confirmed in several studies but remain obscured by considerable variation in measurement and design. A recent systematic review suggested critical gestational periods for maternal cortisol and that the underlying mechanisms may be more complex than simply cortisol levels [54].

In this paper, we focus on three major child outcomes that have been examined in numerous studies to date. The selection of fetal growth, temperament and child mental health is based on the clinical and public health importance of these child developmental outcomes. Robust evidence suggesting an influence on such outcomes from fetal programming mechanisms will have important intervention consequences. Above all, such findings imply that interventions for maternal mental health in pregnancy also need to consider the impact of such conditions of fetal development. Furthermore, effective interventions for both mother and fetus during pregnancy can be considered important prevention interventions for future child development.

In order to select papers of interest we searched Web of Science, Embase, and Scopus Databases, from inception to July 2015, for cohort studies assessing the effects of maternal mental health in pregnancy on the child developmental outcomes of interest. Since the intention was not to conduct a comprehensive systematic review of each outcome, but to provide an overview of recent studies, we were highly selective and focused on studies based on overall study quality and relevance.

### 8.1 Fetal Growth Findings

Fetal growth is a complex process that is regulated by a variety of factors including maternal and fetal genetics, maternal health and nutrition that determines nutrient and oxygen availability to the fetus, maternal endocrine systems and the functioning of the utero-placental system [46,55]. In the majority of epidemiological studies exploring the DOHaD hypothesis, birth size has been used as a proxy for the quality of the intrauterine environment. A number of reviews have already reported consistent associations between exposure to antenatal depression and anxiety and reduced birth size with implications for the growth rate over childhood [6,56].

However, birth size is not an ideal proxy for fetal development since variation of growth within pregnancy is not captured [33,57] and there are several distinct patterns of fetal growth restriction that impact on different parameters of growth. With the advent of routine ultrasound biometry, studies are able to examine fetal growth as a function of maternal mental disorders in pregnancy. To date, nine studies have examined different aspects of the influence of maternal depression, anxiety and stress on fetal growth [32]. Maternal mental health problems experienced during pregnancy have been found to be associated with a variety of adverse fetal growth outcomes including decreased abdominal growth [58], slower fetal weight gain [58,59,60] and reduced fetal head circumference growth [58,59]. Of particular interest are studies that performed multivariate analyses showing that, amongst mental heath symptoms, maternal anxiety is a consistently strong predictor of reductions in fetal head growth. Emerging research also suggests that maternal neuroendocrine function measured via cortisol levels may be a factor mediating the relationship between maternal mental health and fetal development [60,61,62]. However, it is important to place these findings in the context of other deleterious exposures during pregnancy and consider the clinical significance of these exposures for child development. For example, one large study from the Netherlands suggested that the impact of maternal mental health on fetal growth was less than one third that of maternal smoking during pregnancy [59].

A further possible mediating factor between depression, anxiety and fetal growth may be antidepressant exposure, with one study reporting reduced fetal head growth was being associated with SSRI exposure [59]. This study reported that whilst SSRI exposure did reduce depressive symptoms over pregnancy, fetuses exposed to SSRIs also had reduced head growth when compared to the non-medicated groups. The findings are particularly important clinically given that some researchers have suggested that antidepressants may actually protect fetal development from the effects of untreated antenatal depression. Appropriate prescription practices during pregnancy remains a topic of lively debate and require further investigation [23,63].

### 8.2. Temperament

One of the most compelling areas of investigation is the influence of maternal mental health in pregnancy on child temperament. Temperament is considered to reflect individual differences in the regulation of experience and emotions which emerges early in life and remain moderately stable across development [64]. Temperament is distinct from character which develops in a stepwise manner over the life course, progressively assimilating higher-order cognitive capacities, and experience-dependent social and cultural learning, leading to increasingly sophisticated representations of the self over time. Temperament is generally considered to be a highly heritable platform for social and emotional development while conceding that temperament remains open to interaction with the environmental influence across development. As such, the integration of a DOHaD model into studies of the ontogeny of temperament is of great interest.

Several studies have indicated that the infants of mothers with elevated cortisol levels in pregnancy showed increased fussiness and negative behaviour [65]. In a 2007 study by Davis and colleagues mothers who experienced higher late pregnancy cortisol levels reported their infants to show higher levels of negative reactivity. Notably this association remained after controlling for postpartum depression.

Such reports of temperament however are typically based on maternal self-report and so such studies suffer from the potential for bias due to common measurement methods from common informants. So too, it is known that maternal characteristics such as personality and mood can influence maternal ratings of child behaviour [66,67,68,69]. Nonetheless, greater crying, fussing and negative facial expressions in infants of mothers with higher prenatal cortisol levels have also been identified via behavioural observations made by investigators blinded to maternal prenatal variables.

### 8.3. Child Mental Health

Several large cohort studies have examined maternal anxiety and depression in pregnancy to demonstrate prospective prediction of child mental health outcomes. Loomans *et al*. [70] examined prenatal state anxiety and child outcomes at five years in a sample of over 3000 mothers from Amsterdam. Maternal state anxiety measured at 16 weeks gestation was significantly associated with an increased likelihood of inattention/hyperactivity problems for boys (OR = 2.39) but was not significant for girls. In a similar vein, analyses conducted in the Avon Longitudinal Study of Parents and Children [71] study by Connor *et al*. [71,72] in over 7000 mother-child pairs found that prenatal maternal anxiety at 32 weeks was a significant predictor of inattention/hyperactivity symptoms in boys. Clavarino *et al*. [73] examined a sample of close to 4000 and found prenatal maternal anxiety was associated with a moderate increase in risk of attention problems at five that remitted in adolescence and a three-fold increase in risk of anxiety problems that persisted into adolescence. Robinson *et al*. [74] reported data from the *Western Australian Pregnancy Cohort* (*Raine*) *Study* suggesting that pregnancy experiences of major life stressors were associated with an increased likelihood of both emotional and behavioral problems at five years of age.

Very little research has employed the DOHaD framework to examine the child mental health outcomes of pregnant women with severe mental disorders such as bipolar disorder, psychoses, personality disorders, and eating disorders [2]. In an effort to examine the heritability of schizophrenia, there is body of literature following the offspring of pregnant women with schizophrenia, generally indicating that 15% to 40% of children whose parents have schizophrenia develop a psychotic disorder in adulthood [75]. However, much of this work predates the findings on fetal programming mechanisms discussed in this review. In terms of personality disorders, a recent study of a small sample of women diagnosed with Borderline Personality Disorder found high rates of exposure to illicit substances and that a very high proportion of children were referred to child protection services. Women with Borderline Personality Disorder also anticipated the birth of their child as traumatic and frequently requested early delivery. As compared to unaffected infants, the infants born to mothers with Borderline Personality Disorder were significantly more likely to have negative birth outcomes such as lowered Apgar scores, prematurity and to be admitted to a special care nursery [76].

## 9. Conclusions

The current review has found that there is a consistent body of research that suggests that maternal psychological distress, measured mostly as symptoms of depression, anxiety and stress, is a significant factor to be considered in fetal development. Since stress and nutrition are closely interrelated, this suggests that there may be common mechanisms involved. Alteration in stress hormones and changes in specific key nutrients during critical developmental periods may act synergistically to program fetal neurodevelopment [77].

Additional studies with prospective, repeated measure designs that incorporate analysis of known covariates are required to investigate the complex relationship between maternal mental health in pregnancy and fetal development. The field will also be advanced by studies that include measures of the biological mechanisms of this transmission and current investigations of hormonal, epigenetic and placental function are encouraging. Further studies are also needed to further extend this area of research into other forms of maternal psychopathology such as psychotic, bipolar, personality and eating disorders. Efforts to address mental disorders in pregnancy are significant not only in addressing the mother’s wellbeing but increasingly can be understood as a major preventative intervention targeting intrauterine development.

## References

[1] Martins C., Gaffan E.A. (2000). Effects of early maternal depression on patterns of infant-mother attachment: A meta-analytic investigation. J. Child Psychol. Psychiatry.

[2] Stein A., Pearson R.M., Goodman S.H., Rapa E., Rahman A., McCallum M., Howard L.M., Pariante C.M. (2014). Effects of perinatal mental disorders on the fetus and child. Lancet.

[3] Field T. (2011). Prenatal depression effects on early development: A review. Infant Behav. Dev..

[4] Sinclair K., Lea R., Rees W., Young L. (2007). The developmental origins of health and disease: Current theories and epigenetic mechanisms. Soc. Reprod. Fertil. Suppl..

[5] Gluckman P.D., Hanson M.A., Buklijas T. (2010). A conceptual framework for the developmental origins of health and disease. J. Dev. Orig. Health Dis..

[6] Ding X.-X., Wu Y.-L., Xu S.-J., Zhu R.-P., Jia X.-M., Zhang S.-F., Huang K., Zhu P., Hao J.H., Tao F.B. (2014). Maternal anxiety during pregnancy and adverse birth outcomes: A systematic review and meta-analysis of prospective cohort studies. J. Affect. Disord..

[7] Galbally M., Snellen M., Lewis A.J. (2011). A review of the use of psychotropic medication in pregnancy. Curr. Opin. Obstet. Gynecol..

[8] Buist A., Bilszta J. (2006). The beyondblue National Postnatal Screening Program, Prevention and Early Intervention 2001–2005, Final Report Volume I: National Screening Program.

[9] Bennett H.A., Einarson A., Taddio A., Koren G., Einarson T.R. (2004). Prevalence of depression during pregnancy: Systematic review. Obstet. Gynecol..

[10] Gotlib I.H., Whiffen V.E., Mount J.H., Milne K., Cordy N.I. (1989). Prevalence rates and demographic characteristics associated with depression in pregnancy and the postpartum. J. Consult. Clin. Psychol..

[11] Andersson L., Sundström-Poromaa I., Bixo M., Wulff M., Bondestam K., Åstöm M. (2003). Point prevalence of psychiatric disorders during the second trimester of pregnancy: A population-based study. Am. J. Obstet. Gynecol..

[12] Howard L.M., Molyneaux E., Dennis C.-L., Rochat T., Stein A., Milgrom J. (2014). Non-psychotic mental disorders in the perinatal period. Lancet.

[13] Lewis A.J., Bailey C., Galbally M. (2012). Anti-depressant use during pregnancy in Australia: Findings from the Longitudinal Study of Australian Children. Aust. N. Z. J. Public Health.

[14] Suzuki K., Sato M., Zheng W., Shinohara R., Yokomichi H., Yamagata Z. (2014). Effect of maternal smoking cessation before and during early pregnancy on fetal and childhood growth. J. Epidemiol..

[15] Novakovic B., Ryan J., Pereira N., Boughton B., Craig J.M., Saffery R. (2014). Postnatal stability, tissue, and time specific effects of AHRR methylation change in response to maternal smoking in pregnancy. Epigenetics.

[16] Behnke M., Smith V.C. (2013). Prenatal substance abuse: Short- and long-term effects on the exposed fetus. Pediatrics.

[17] Hoffman J.D. (2011). Pregnancy and Alcohol Consumption.

[18] Carter R.C., Jacobson J.L., Sokol R.J., Avison M.J., Jacobson S.W. (2013). Fetal alcohol-related growth restriction from birth through young adulthood and moderating effects of maternal prepregnancy weight. Alcohol. Clin. Exp. Res..

[19] Auttiramo I., Gaily E., Granstrom M.L. (1992). Dysmorphic features in offspring of alcoholic mothers. Arch. Dis. Child..

[20] Hingson R., Alpert J.J., Day N., Dooling E., Kayne H., Morelock S., Oppenheimer E., Zuckerman B. (1982). Effects of Maternal drinking and marijuana use on fetal growth and development. Pediatrics.

[21] Galbally M., Gentile S., Lewis A.J. (2012). Further findings linking SSRIs during pregnancy and persistent pulmonary hypertension of the newborn: Clinical implications. CNS Drugs.

[22] Galbally M., Lewis A.J. (2013). Prenatal exposure to antidepressants is associated with small but significant differences in pregnancy and delivery outcomes. Evid. Based Ment. Health.

[23] Galbally M., Lewis A.J., Buist A. (2011). Developmental outcomes of children exposed to antidepressants in pregnancy. Aust. N. Z. J. Psychiatry.

[24] Galbally M., Lewis A., Lum J., Buist A. (2009). Serotonin discontinuation syndrome following in utero exposure to antidepressant medication: Prospective controlled study. Aust. N. Z. J. Psychiatry.

[25] Lewis A.J., Galbally M. (2011). Review: Adverse effects of antidepressants use during pregnancy. Evid. Based Ment. Health.

[26] Lewis A.J., Galbally M., Opie G., Buist A. (2010). Neonatal growth outcomes at birth and one month postpartum following in utero exposure to antidepressant medication. Aust. N. Z. J. Psychiatry.

[27] Andrade S.E., Raebel M.A., Brown J., Lane K., Livingston J., Boudreau D., Rolnick S.J., Roblin D., Smith D.H., Willy M.E. (2008). Use of antidepressant medications during pregnancy: A multisite study. Am. J. Obstet. Gynecol..

[28] Rodriguez-Bernal C.L., Rebagliato M., Ballester F. (2012). Maternal nutrition and fetal growth: The role of iron status and intake during pregnancy. Nutr. Diet. Suppl..

[29] Robinson M., Whitehouse A.J.O., Newnham J.P., Gorman S., Jacoby P., Holt B.J., Serralha M., Tearne J.E., Holt P.G., Hart P.H. (2014). Low maternal serum vitamin D during pregnancy and the risk for postpartum depression symptoms. Arch. Womens Ment. Health.

[30] Wu G.Y., Imhoff-Kunsch B., Girard A.W. (2012). Biological mechanisms for nutritional regulation of maternal health and fetal development. Paediatr. Perinat. Epidemiol..

[31] Parsons T.J., Power C., Manor O. (2001). Fetal and early life growth and body mass index from birth to early adulthood in 1958 British cohort: Longitudinal study. BMJ.

[32] Lewis A.J., Austin E., Galbally M. (2015). Maternal mental health and fetal growth restriction: A systematic review. J. Dev. Orig. Health Dis..

[33] Lewis A.J. (2014). Depression in Pregnancy and Child Development: Understanding the Mechanisms of Transmission.

[34] Lerner R.M. (2001). Concepts and Theories of Human Development.

[35] Rutter M., Sroufe L. (2000). Developmental psychopathology: Concepts and challenges. Dev. Psychopathol..

[36] Barker D.J.P. (1995). The Wellcome Foundation Lecture, 1994. The fetal origins of adult disease. Proc. Biol. Sci..

[37] Lewis A.J. (2012). A call for an expanded synthesis of developmental and evolutionary paradigms. Behav. Brain Sci..

[38] Lewis A.J., Galbally M., Gannon T., Symeonides C. (2014). Early life programming as a target for prevention of child and adolescent mental disorders. BMC Med..

[39] Leung C.Y., Leung G.M., Schooling C.M. (2015). Early second-hand smoke exposure and child and adolescent mental health: Evidence from Hong Kong’s “Children of 1997” birth cohort. Addiction.

[40] Bertram C.E., Hanson M.A. (2002). Prenatal programming of postnatal endocrine responses by glucocorticoids. Reproduction.

[41] Meaney M.J. (2010). Epigenetics and the biological definition of gene × environment interactions. Child Dev..

[42] Galbally M., Lewis A.J., Ijzendoorn M., Permezel M. (2011). The role of oxytocin in mother-infant relations: A systematic review of human studies. Harv. Rev. Psychiatry.

[43] Evans-Galea M.V., Hannan A.J., Carrodus N., Delatycki M.B., Saffery R. (2013). Epigenetic modifications in trinucleotide repeat diseases. Trends Mol. Med..

[44] Bird A. (2007). Perceptions of epigenetics. Nature.

[45] O’Donnell K.J., Bugge Jensen A., Freeman L., Khalife N., O’Connor T.G., Glover V. (2012). Maternal prenatal anxiety and downregulation of placental 11β-HSD2. Psychoneuroendocrinology.

[46] Owens J.A. (1991). Endocrine and substrate control of fetal growth: Placental and maternal influences and insulin-like growth factors. Reprod. Fertil. Dev..

[47] Barker D.J. (1997). The long-term outcome of retarded fetal growth. Clin. Obstet. Gynecol..

[48] Massaro A.N., Rothbaum R., Aly H. (2006). Fetal brain development: The role of maternal nutrition, exposures and behaviors. J. Pediatr. Neurol..

[49] Seth S., Lewis A.J., Saffery R., Lappas M., Galbally M. (2015). Maternal prenatal mental health and placental 11b-HSD2 gene expression: Initial findings from the mercy pregnancy and emotional wellbeing study. Int. J. Mol. Sci..

[50] Seckl J.R. (2004). Prenatal glucocorticoids and long-term programming. Eur. J. Endocrinol..

[51] Seng J.S., Rauch S.A.M., Resnick H., Reed C.D., King A., Low L.K., McPherson M., Muzik M., Abelson J., Liberzon I. (2010). Exploring posttraumatic stress disorder symptom profile among pregnant women. J. Psychosom. Obstet. Gynaecol..

[52] Robinson J., Chidzanja S., Kind K., Lok F., Owens P., Owens J. (1995). Placental control of fetal growth. Reprod. Fertil. Dev..

[53] Mulder E.J.H., de Medina P.G.R., Huizink A.C., van den Bergh B.R.H., Buitelaar J.K., Visser G.H.A. (2002). Prenatal maternal stress: Effects on pregnancy and the (unborn) child. Early Hum. Dev..

[54] Zijlmans M.A.C., Riksen-Walraven J.M., de Weerth C. (2015). Associations between maternal prenatal cortisol concentrations and child outcomes: A systematic review. Neurosci. Biobehav. Rev..

[55] Gicquel C., le Bouc Y. (2006). Hormonal regulation of fetal growth. Horm. Res..

[56] Field T., Diego M., Hernandez-Reif M. (2006). Prenatal depression effects on the fetus and newborn: A review. Infant Behav. Dev..

[57] Simmons R. (2005). Developmental origins of adult metabolic disease: Concepts and contraversies. Trends Endocrinol. Metab..

[58] Henrichs J., Schenk J.J., Roza S.J., van den Berg M.P., Schmidt H.G., Steegers E.A., Hofman A., Jaddoe V.W., Verhulst F.C., Tiemeier H. (2010). Maternal psychological distress and fetal growth trajectories: The generation R study. Psychol. Med..

[59] El Marroun H., Jaddoe V.W., Hudziak J.J., Roza S.J., Steegers E.A., Hofman A., Verhulst F.C., White T.J., Stricker B.H., Tiemeier H. (2012). Maternal use of selective serotonin reuptake inhibitors, fetal growth, and risk of adverse birth outcomes. Arch. Gen. Psychiatry.

[60] Diego M.A., Field T., Hernandez-Reif M., Schanberg S., Kuhn C., Gonzalez-Quintero V.H. (2009). Prenatal depression restricts fetal growth. Early Hum. Dev..

[61] Diego M.A., Jones N.A., Field T., Hernandez-Reif M., Schanberg S., Gonzalez-Garcia A., Kuhn C. (2006). Maternal psychological distress, prenatal cortisol, and fetal weight. Psychosom. Med..

[62] Field T., Diego M.A., Hernandez-Reif M., Figueiredo B., Ascencio A., Schanberg S., Kuhn C. (2008). Prenatal dysthymia *versus* major depression effects on maternal cortisol and fetal growth. Depress. Anxiety.

[63] Oberlander T.F. (2012). Fetal serotonin signaling: Setting pathways for early childhood development and behavior. J. Adolesc. Health.

[64] Lewis A.J., Olsson C.A. (2011). Early life stress and child temperament style as predictors of childhood anxiety and depressive symptoms: Findings from the longitudinal study of Australian children. Depress. Res. Treat..

[65] Davis E.P., Glynn L.M., Schetter C.D., Hobel C., Chicz-Demet A., Sandman C.A. (2007). Prenatal exposure to maternal depression and cortisol influences infant temperament. J. Am. Acad. Child Adolesc. Psychiatry.

[66] Goodman S.H., Rouse M.H., Connell A.M., Broth M.R., Hall C.M., Heyward D. (2011). Maternal depression and child psychopathology: A meta-analytic review. Clin. Child Fam. Psychol. Rev..

[67] Fergusson D.M., Lynskey M.T., Horwood L.J. (1993). The effect of maternal depression on maternal ratings of child behavior. J. Abnorm. Child Psychol..

[68] Durbin C.E., Wilson S. (2012). Convergent validity of and bias in maternal reports of child emotion. Psychol. Assess..

[69] Lewis A.J., Bertino M.D., Bailey C.M., Skewes J., Lubman D.I., Toumbourou J.W. (2014). Depression and suicidal behavior in adolescents: A multi-informant and multi-methods approach to diagnostic classification. Front. Psychol..

[70] Loomans E.M., van der Stelt O., van Eijsden M., Gemke R.J.B.J., Vrijkotte T., van den Bergh B.R. (2011). Antenatal maternal anxiety is associated with problem behaviour at age five. Early Hum. Dev..

[71] O’Connor T.G., Heron J., Golding J., Glover V., the ALSPAC Study Team (2003). Maternal antenatal anxiety and behavioural/emotional problems in children: A test of a programming hypothesis. J. Child Psychol. Psychiatry.

[72] O’Connor T.G., Heron J., Golding J., Beveridge M., Glover V. (2002). Maternal antenatal anxiety and children’s behavioural/emotional problems at 4 years: Report from the Avon Longitudinal Study of Parents and Children. Br. J. Psychiatry.

[73] Clavarino A.M., Mamun A.A., O’Callaghan M., Aird R., Bor W., O’Callaghan F., Williams G.M., Marrington S., Najman J.M., Alati R. (2010). Maternal anxiety and attention problems in children at 5 and 14 years. J. Atten. Disord..

[74] Robinson M., Oddy W.H., Li J., Kendall G.E., de Klerk N.H., Silburn S.R., Zubrick S.R., Newnham J.P., Stanley F.J., Mattes E. (2008). Pre- and postnatal influences on preschool mental health: A large-scale cohort study. J. Child Psychol. Psychiatry.

[75] Hammed M., Lewis A.J. (2015). Offspring of parents with schizophrenia: A systematic review of developmental features across childhood. Harv. Rev. Psychiatry.

[76] Blankley G., Galbally M., Snellen M., Power J., Lewis A.J. (2015). Borderline personality disorder in the perinatal period: Outcomes for mothers and infants. Australas. Psychiatry.

[77] Huizink A.C., Mulder E.J.H., Buitelaar J.K. (2004). Prenatal stress and risk for psychopathology: Specific effects or induction of general susceptibility?. Psychol. Bull..

